# African Arrow Poisons

**Published:** 1916-04

**Authors:** 


**African Arrow Poisons.** Castellanix and Chälmer's Manual of Tropical Medicine is quoted (ibid.) to the effect that these poisons are made by evaporating a decoction of various species of acocanthera, many tribes adding snake's heads and gall bladders which probably do not add to the toxicity, although symptoms of venom poisoning are occasionally noted. Treatment consists in ligation to prevent absorption of poison, removal of arrow head, which is usually barbed, prompt lavage with potassium permanganate, stimulation of heart, cupping or sucking of wound, the after treatment consisting in the ordinary routine of a poisoned wound, antitetanic serum being advised on general principles. Death in untreated wounds occurs within a few minutes by cardiac failure, after a brief period of quickened respiration and convulsions. The potassium permanganate method is regarded as efficacious if promptly applied. Natives are supposed to immunize themselves by eating the poisonous plants themselves. (Note: It is interesting to learn that the profession in S. Africa is still meeting problems of the early part of the 19th century and of the 18th and 17th in America, though arrow poisons were scarcely used by the northern American Indians.—Ed.)

